# Context-Dependent Roles for SIRT2 and SIRT3 in Tumor Development Upon Calorie Restriction or High Fat Diet

**DOI:** 10.3389/fonc.2019.01462

**Published:** 2020-01-08

**Authors:** Mohamed A. Ahmed, Carol O'Callaghan, Elliot D. Chang, Haiyan Jiang, Athanassios Vassilopoulos

**Affiliations:** ^1^Department of Radiation Oncology, Feinberg School of Medicine, Northwestern University, Chicago, IL, United States; ^2^Radiation Biology Department, National Center for Radiation Research and Technology, Cairo, Egypt; ^3^Robert H. Lurie Comprehensive Cancer Center, Feinberg School of Medicine, Northwestern University, Chicago, IL, United States

**Keywords:** SIRT2, SIRT3, calorie restriction, high fat diet, cancer, aging

## Abstract

Calorie restriction (CR) is considered one of the most robust ways to extend life span and reduce the risk of age-related diseases, including cancer, as shown in many different organisms, whereas opposite effects have been associated with high fat diets (HFDs). Despite the proven contribution of sirtuins in mediating the effects of CR in longevity, the involvement of these nutrient sensors, specifically, in the diet-induced effects on tumorigenesis has yet to be elucidated. Previous studies focusing on SIRT1, do not support a critical role for this sirtuin family member in CR-mediated cancer prevention. However, the contribution of other family members which exhibit strong deacetylase activity is unexplored. To fill this gap, we aimed at investigating the role of SIRT2 and SIRT3 in mediating the anti and pro-tumorigenic effect of CR and HFD, respectively. Our results provide strong evidence supporting distinct, context-dependent roles played by these two family members. SIRT2 is indispensable for the protective effect of CR against tumorigenesis. On the contrary, SIRT3 exhibited oncogenic properties in the context of HFD-induced tumorigenesis, suggesting that SIRT3 inhibition may mitigate the cancer-promoting effects of HFD. Given the different functions regulated by SIRT2 and SIRT3, unraveling downstream targets/pathways involved may provide opportunities to develop new strategies for cancer prevention.

## Introduction

Calorie restriction (CR); 20-40% reduction of daily calorie intake without malnutrition, is the most studied and reproducible non-genetic intervention known to extend lifespan in different species (1–8). In addition to positively affecting longevity, CR improves also healthspan as evidenced by the reduced risk for a variety of age-associated diseases, including diabetes, sarcopenia, cardiovascular diseases, and hearing loss in both rodents and humans (9). The opposite effect is exerted by obesity and high-fat diets. In this regard, the high-fat diet (HFD)-fed mouse has been well-established as a model for impaired glucose tolerance (IGT) and type 2 diabetes (10). In a similar way, obesity in humans is linked with increased mortality due to several pathologies and major reductions in life expectancy compared with normal weight (11).

Given that cancer is considered an age-related disease (12), any interventions shown to improve healthspan could be exploited to either prevent or slow down tumorigenesis. Indeed, CR delays tumorigenesis in a *p53*-knockout mouse model (13) and recent studies show a significant reduction in the incidence of cancers in rhesus monkeys fed a CR diet compared to a control diet (14). Accordingly, a recent meta-analysis showed that 40 of the 44 pre-clenical studies (90.9%) support the anticancer role of CR despite the different measurements (15). On the contrary, HFD-induced obesity was found to shorten life expectancy through mediating various diseases, including cancer (16–18). While the anti and pro-tumorigenic effects of CR and HFD, respectively, are well-established, the underlying mechanisms remain obscure.

Considering that sirtuins mediate the CR-induced anti-aging effects (19, 20) through their function as NAD-dependent protein deacetylases directly activated by CR, it could be proposed that they may contribute to the tumor suppressive effects of CR. Despite extensive research regarding the broader role of sirtuins in cancer (21), this is a relatively unexplored area. Therefore, it is rather surprising that there is a lack of experimental data to assess the involvement of sirtuins, specifically, in the CR-induced effect on tumorigenesis. Among the different family members, SIRT1, is the only sirtuin studied so far in this context. More specifically, its overexpression failed to influence the anticancer effects of every-other-day fasting-a variation of CR–(22). Although these result suggest that SIRT1 may play a limited role in the effects of CR on cancer, the possible involvement of other sirtuins in the diet-induced effects on tumorigenesis has yet to be elucidated.

SIRT2 and SIRT3 exhibit the strongest deacetylation activity compared to other family members (23) and have been shown to regulate the acetylome (24, 25). Here, we investigated the role of these two family members in mediating the anti and pro-tumorigenic effect of CR and HFD, respectively. Toward this direction, heterozygous^(+/−)^ and homozygous^(−/−)^ *p53* deleted C57BL/6 mice were crossed with either *Sirt2*^−/−^ or *Sirt3*^−/−^ mice to generate mice with 6 different genotypes: *p53*^+/−^, *Sirt2*^−/−^; *p53*^+/−^, *Sirt3*^−/−^; *p53*^+/−^, *p53*^−/−^, *Sirt2*^−/−^; *p53*^−/−^, and *Sirt3*^−/−^; *p53*^−/−^. Each of the 6 genotypes was subjected to 3 dietary regimes; *ad libitum* control diet, 30% CR diet, and *ad libitum* HFD, starting at about 7-8 weeks after birth followed by survival and tumor incidence analysis.

## Materials and Methods

### Mice

p53 knockout mice [Stock No. 002101, The Jackson Laboratory, (26)] were kindly provided by Elizabeth Eklund (Northwestern University). *Sirt2*^−/−^ and *Sirt3*^−/−^ mice have been generated and described previously (27, 28). Hemizygous (+/-) and nullizygous (-/-) *p53* mice were crossed with either *Sirt2*^−/−^ or *Sirt3*^−/−^ mice to generate mice with 6 different genotypes: *p53*^+/−^, *Sirt2*^−/−^; *p53*^+/−^, *Sirt3*^−/−^; *p53*^+/−^, *p53*^−/−^, *Sirt2*^−/−^; *p53*^−/−^, and *Sirt3*^−/−^; *p53*^−/−^. Mice were housed and treated in accordance with the guidelines approved by the Northwestern University Institutional Animal Care and Use Committee (IACUC).

### Genotyping

Mouse genomic DNA was isolated from tail biopsies using the REDExtract-N-Amp™ Tissue PCR Kit following the standard protocol. PCR was performed using the primer pairs and conditions as described below:

For p53
Forward wild-type 5′-AGGCTTAGAGGTGCAAGCTG-3′,
Forward mutant 5′-CAGCCTCTGTTCCACATACACT-3′,
Reverse (common) 5′-TGGATGGTGGTATACTCAGAGC-3′
32 cycles at 93°C for 30 s, 60°C for 30 s, and 65°C for 2 min.
For SIRT2
Forward wild-type 5′-CAGGGTCTCACGAGTCTCATG-3′,
Forward mutant 5′-GACTGGAAGTGATCAAAGCTC-3′,
Reverse (common) 5′-TCAAATCTGGCCAGAACTTATG-3′
35 cycles at 94 °C for 30 s, 55°C for 30 s, and 72°C for 60 s.
For SIRT3
Forward (common) 5′-GGGAGCACTCTCATACTCTA-3′,
Reverse wild-type 5′-TTACTGCTGCCTAACGTTCC-3′
Reverse mutant 5′-CCCTCAATCACAAATGTCGG-3′,
32 cycles at 94°C for 10 s, 60°C for 20 s, and 72°C for 30 s.

### Diets

Each of the 6 genotypes (*n* = 9–12) was subjected to 3 dietary regimes; *ad libitum* control diet (3.85 kcal/g, D12450Ji), 30% calorie restriction diet (D15032801Bi), and *ad libitum* high-fat diet (5.24 kcal/g with 60% kcal from fat, D12492Ri), starting at about 7-8 weeks after birth. Diets were purchased from Research Diet Inc. (Brunswick, NJ, USA), and were designed to provide variations in calories while maintaining similar micronutrient composition. Heterozygous *p53* knockout mice were subjected to two cycles of either CR or HFD-14 weeks each, intercepted by 21 weeks of exposure to control diet pellets. Homozygous *p53* knockout mice displayed shorter lifespan (~60% shorter than *p53* heterozygous knockout mice), and were subjected to one 14 weeks long cycle of either CR or HFD.

### Body Weight, Tumor Incidence, and Survival

Diets were administered by members of the Center for Comparative Medicine (CCM) at Northwestern University. Body weights and health condition of the mice were closely monitored and reports were provided to the lab members. All data were analyzed by lab members. Tumors and tissues were collected at the end of the experiments.

### Western Blotting

Tissues were homogenized in cold lysis buffer (50 mM Tris-HCl, pH 7.5, 150 mM NaCl, 1% NP-40), with freshly added protease and phosphatase inhibitors. The following antibodies were used: SIRT2 (Proteintech, #19655-1-AP), SIRT3 (Cell Signaling, #5490), p53 (1C12) (Cell Signaling, #2524S), and HRP-conjugated beta Actin (Proteintech, #HRP-60008). Antibody detection was accomplished using HRP-conjugated secondary antibodies, and the chemiluminescence signals developed after incubation with ECL (Azure Biosystems) were detected using C-400 imaging system (Azure Biosystems).

### Statistical Analysis

All graphs, statistical analysis, and calculation of *p*-values were carried out using GraphPad Prism software (GraphPad Software Inc., San Diego, CA, USA). Overall survival and tumor-free survival were plotted by Kaplan-Meier curves and analyzed using the log-rank (Mantel-Cox) test. The hazard ratios, indicating changes in tumor incidence rate, were calculated from the slopes of tumor-free survival curves and estimated based on log-rank test.

## Results

### Effect of SIRT2 and SIRT3 Loss on Body Weight Upon CR and HFD

**Figure 1:**
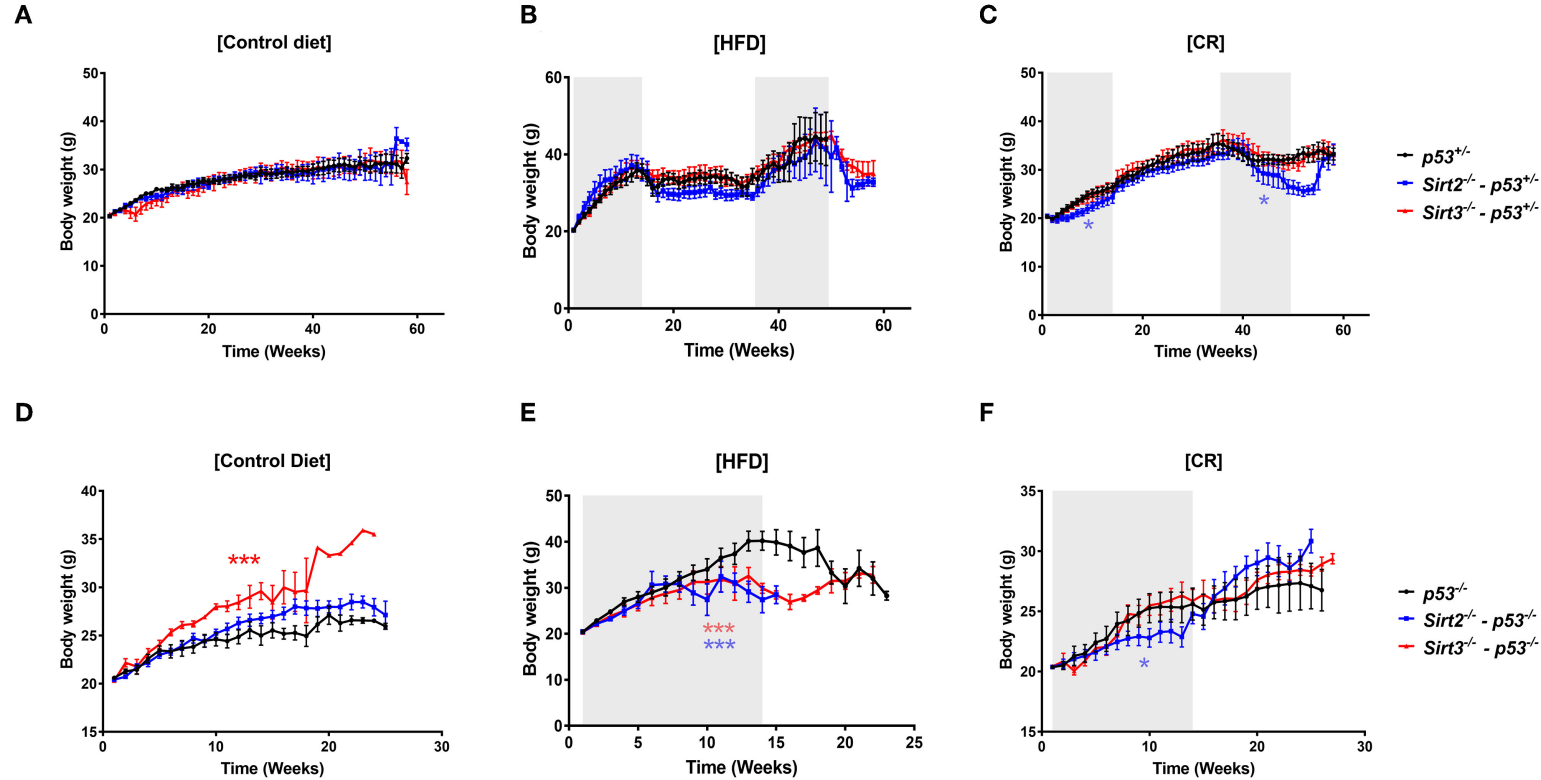
Body weight changes in response to the different diets. Body weights of *Sirt2*^−/−^ (blue line), *Sirt3*^−/−^ (red line), and control *Sirt2*^*wt*^/*Sirt3*^*wt*^ (black line) mice heterozygous or nullizygous for *p53* were measured weekly during exposure to **(A,D)** a control diet, **(B,E)** a high fat diet, or **(C,F)** a 30% calorie restricted diet. *p < 0.05, ***p < 0.001.

No change in body weight of *Sirt2*^−/−^ and *Sirt3*^−/−^ mice fed on control diet was observed, as compared to control mice with the exception of *Sirt3*^−/−^*; p53*^−/−^ mice which exhibited increased body weight (Figures 1A,D). Upon CR, all mice responded to the diet as evidenced by the decrease in body weight and the weight gain following withdrawal from calorie restriction (Figures 1C,F). Of note, *Sirt2*^−/−^ mice appeared to show the greatest effect, based on the significant decreased body weight compared to other mice (Figures 1C,F). On the other hand, *Sirt3*^−/−^ mice were indistinguishable compared to wild-type (Figures 1C,F) with regards to body weight following CR. This finding is consistent with previous reports showing no difference in the body weight of *Sirt3* deleted mice compared to *Sirt3* wild-type ones under CR conditions (29, 30). Upon HFD, although a similar gain weight was observed in all mice in the *p53*^+/−^ background (Figure 1B), both *Sirt2*^−/−^ and *Sirt3*^−/−^ mice in the *p53*^−/−^ background displayed decreased body weights as compared to control mice (Figure 1E).

### SIRT2 Deletion Abolishes the Cancer-Preventive Effect of CR

**Figure 2:**
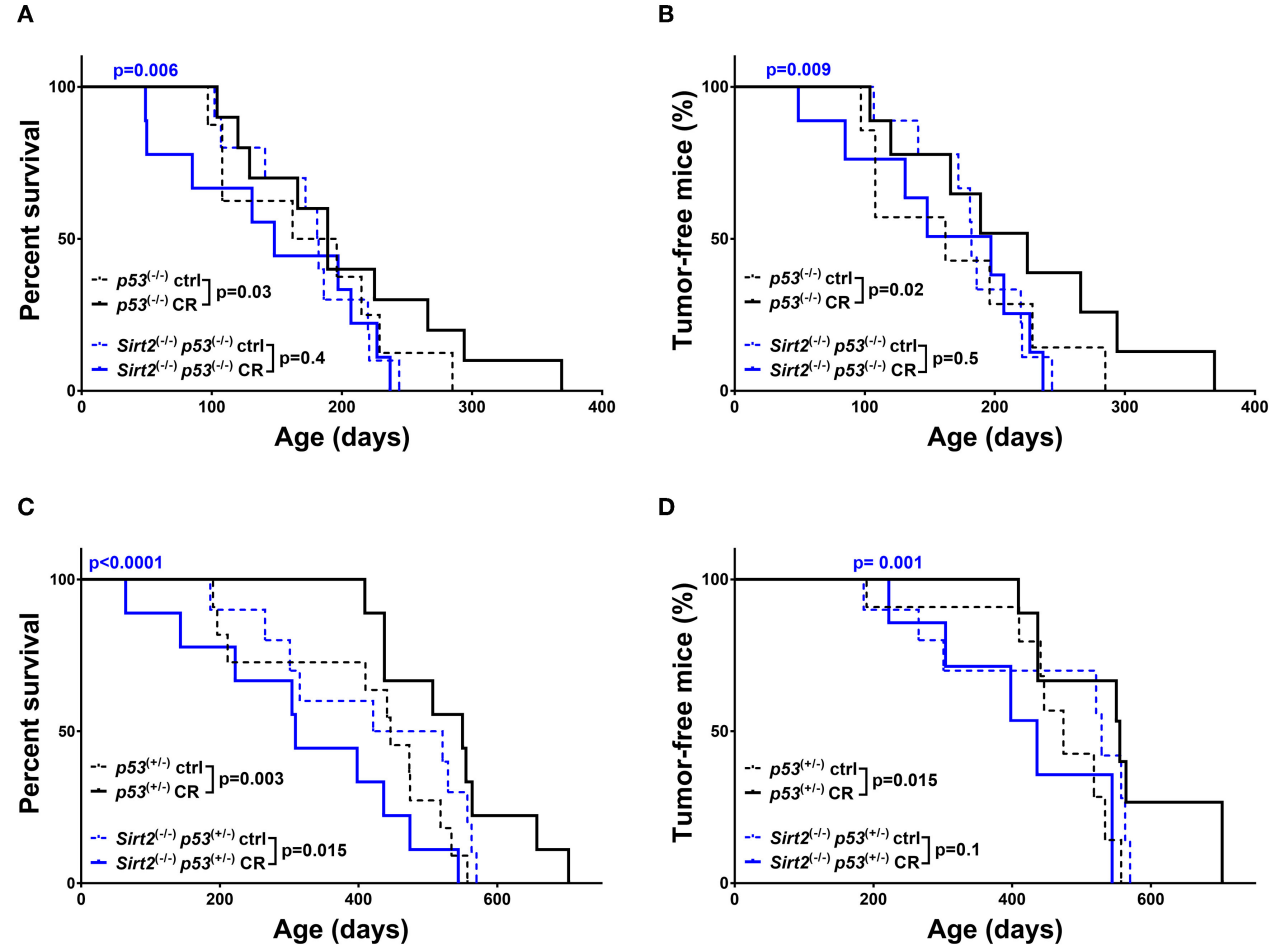
*Sirt2* deficiency abolished CR-mediated increased survival and tumor protection in mice. Kaplan-Meier curves show overall survival **(A,C)** and tumor free survival **(B,D)**. *Sirt2*^−/−^*; p53*^−/−^ (blue lines) and *p53*^−/−^ (black lines) mice **(A,B)**, *Sirt2*^−/−^*; p53*^+/−^ (blue lines) and *p53*^+/−^ (black lines) **(C,D)** were included in the analysis. Mice were either calorie restricted (CR) or fed *ad libitum* control diet. Solid lines represent mice in the CR groups, whereas control diet groups are represented in dotted lines. The blue *p*-values on the top of each curve represent the statistical difference in survival and tumor incidence between calorie restricted *Sirt2*^−/−^ mice, and calorie restricted *Sirt2*^+/+^ mice with similar *p53 g*enotypes. All *p*-values are calculated using the log rank test.

Consistent with previous studies (13, 31), CR exerted a beneficial effect in both *p53*^+/−^ and *p53*^−/−^ mice (Figure 2 and Supplementary Table 1). This is reflected by increased overall survival (*p53*^+/−^: CR vs. ctrl *p* = 0.003 / *p53*^−/−^: CR vs. ctrl *p* = 0.03) and median survival (*p53*^+/−^: 446 days ctrl vs. 550 days CR/*p53*^−/−^: 179 days ctrl vs. 189 days CR), with concomitant significant decrease in tumor incidence (*p53*^+/−^: CR vs. ctrl *p* = 0.015-HR = 0.46/*p53*^−/−^: CR vs. ctrl *p* = 0.02-HR = 0.55, Figure 2 and Supplementary Table 1). Of note, deletion of *Sirt2* in both *p53*^+/−^ and *p53*^−/−^ backgrounds abolished the protective effect of CR. In both the *Sirt2*^−/−^; *p53*^−/−^ (Figures 2A,B) and *Sirt2*^−/−^; *p53*^+/−^ (Figures 2C,D) mice, the beneficial effect of CR as compared to the control diet was not observed based on the overall survival (*Sirt2*^−/−^; *p53*^−/−^: CR vs. ctrl *p* = 0.4 / *Sirt2*^−/−^; *p53*^+/−^: CR vs. ctrl *p* = 0.015) and the tumor incidence (*Sirt2*^−/−^; *p53*^−/−^: CR vs. ctrl *p* = 0.5/*Sirt2*^−/−^; *p53*^+/−^: CR vs. ctrl *p* = 0.1). This happened despite the decreased overall weight of calorie restricted *Sirt2*^−/−^ mice as compared to *Sirt2* wild-type mice (Figures 1C,F). Consistently, calorie restricted *Sirt2*^−/−^; *p53*^−/−^*;* and *Sirt2*^−/−^; *p53*^+/−^ compared to *p53*^−/−^ and *p53*^+/−^ mice showed significantly decreased overall survival (CR: *Sirt2*^−/−^; *p53*^−/−^ vs. *p53*^−/−^*p* = 0.006 / *Sirt2*^−/−^; *p53*^+/−^ vs. *p53*^+/−^*p* < 0.0001) and increased tumor incidence (CR: *Sirt2*^−/−^; *p53*^−/−^ vs. *p53*^−/−^*p* = 0.009 / *Sirt2*^−/−^; *p53*^+/−^ vs. *p53*^+/−^*p* = 0.001).

**Figure 3:**
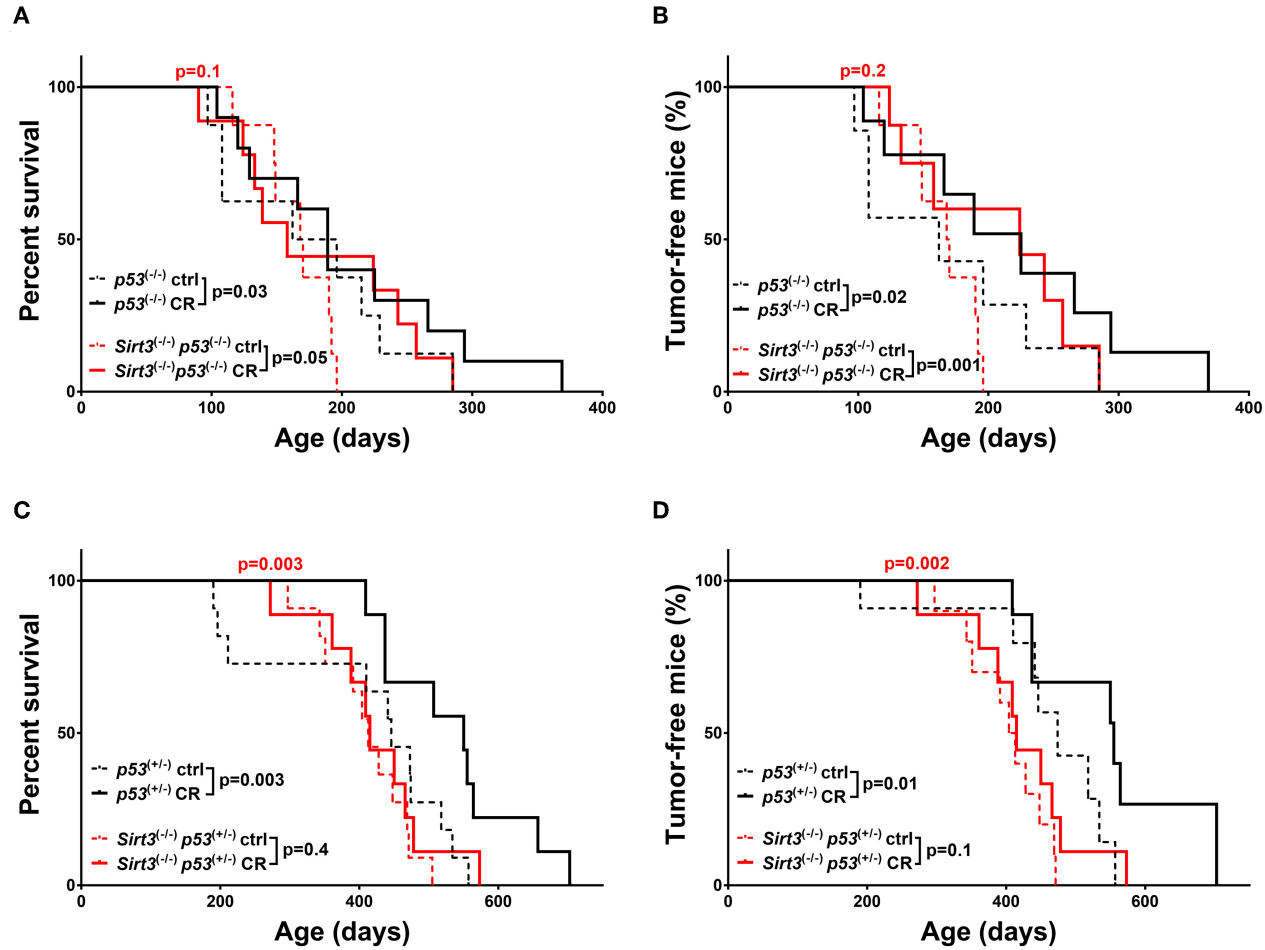
Overall survival and tumor incidence in calorie-restricted *Sirt3*^−/−^ mice. Kaplan-Meier curves show overall survival **(A,C)** and tumor free survival **(B,D)**. *Sirt3*^−/−^*; p53*^−/−^ (red lines) and *p53*^−/−^ (black lines) mice **(A,B)**, *Sirt3*^−/−^*; p53*^+/−^ (red lines) and *p53*^+/−^ (black lines) **(C,D)** were included in the analysis. Mice were either calorie restricted (CR) or fed *ad libitum* control diet. Solid lines represent mice in the CR groups, whereas control diet groups are represented in dotted lines. The red *p*-values on the top of each curve represent the statistical difference in survival and tumor incidence between calorie restricted *Sirt3*^−/−^ mice, and calorie restricted *Sirt3*^+/+^ mice with similar *p53 g*enotypes. All *p*-values are calculated using the log rank test.

On the contrary, the beneficial effect from CR was maintained in the *Sirt3*^−/−^*; p53*^−/−^ mice as evidenced by the increased survival (*Sirt3*^−/−^*; p53*^−/−^: CR vs. ctrl *p* = 0.05) and delay in tumor development (*Sirt3*^−/−^*; p53*^−/−^: CR vs. ctrl *p* = 0.001) (Figures 3A,B). Accordingly, survival and tumor incidence curves upon CR were identical in *Sirt3*^−/−^*; p53*^−/−^*;* and *p53*^−/−^ mice suggesting that both benefited (CR: *Sirt3*^−/−^; *p53*^−/−^ vs. *p53*^−/−^*p* = 0.1 survival/CR: *Sirt3*^−/−^; *p53*^−/−^ vs. *p53*^−/−^*p* = 0.2 tumor incidence). Interestingly, this was not the case in the *p53* heterozygous background, where *Sirt3*^−/−^; *p53*^+/−^ mice did not benefit from CR (*Sirt3*^−/−^*p53*^+/−^: CR vs. ctrl *p* = 0.4 survival / CR vs. ctrl *p* = 0.1 tumor incidence) (Figures 3C,D and Supplementary Table 1). It is worth mentioning that *p53*^+/−^ control diet-fed mice deleted for *Sirt3* exhibited accelerated tumor incidence when compared to *Sirt3* wild-type mice, (Figure 3D) which could possibly mask any benefits from CR. All together, our data suggest that among the two family members studied here, SIRT2 exhibits strong tumor suppressive properties upon CR regardless of the *p53* genetic background, consistent with a role for SIRT2 in mediating the cancer preventive effect of CR.

### SIRT3 Deletion Renders Mice Resistant to HFD-Induced Tumor Development

**Figure 4:**
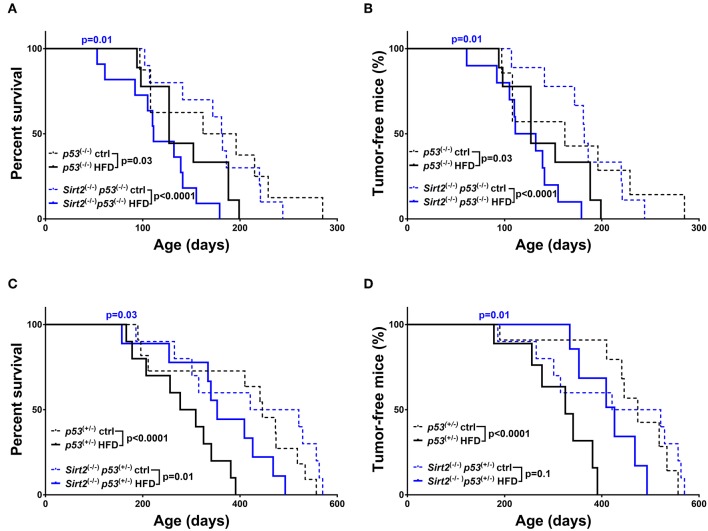
Effect of *Sirt2* deletion in overall survival and tumorigenesis upon HFD. Kaplan-Meier curves show overall survival **(A,C)** and tumor free survival **(B,D)**. *Sirt2*^−/−^*; p53*^−/−^ (blue lines) and *p53*^−/−^ (black lines) mice **(A,B)**, *Sirt2*^−/−^*; p53*^+/−^ (blue lines) and *p53*^+/−^ (black lines) **(C,D)** were included in the analysis. Mice were either fed a high fat diet (HFD) or fed *ad libitum* control diet. Solid lines represent mice in the HFD groups, whereas control diet groups are represented in dotted lines. The blue *p*-values on the top of each curve represent the statistical difference in survival and tumor incidence between HFD *Sirt2*^−/−^ mice, and HFD *Sirt2*^+/+^ mice with similar *p53 g*enotypes. All *p*-values are calculated using the log rank test.

As already mentioned before, obesity, and high-fat diets exert the complete opposite effects compared to caloric restriction. Accordingly, both *p53*^+/−^ and *p53*^−/−^ mice fed on a HFD exhibited a significant decrease in overall survival (*p53*^+/−^: HFD vs. ctrl *p* < 0.0001 / *p53*^−/−^: HFD vs. ctrl *p* = 0.03) and median survival (*p53*^+/−^: 446 days ctrl vs. 293 days HFD / *p53*^−/−^:179 days ctrl vs. 139.5 days HFD) which was further associated with increased tumor incidence (*p53*^+/−^: HFD vs. ctrl *p* < 0.0001, HR = 4.01 / *p53*^−/−^: HFD vs. ctrl *p* = 0.03, HR = 1.58), as compared to the control diet group (Figure 4 and Supplementary Table 1). The detrimental effects of HFD were potentiated in *Sirt2*^−/−^*; p53*^−/−^ mice after analyzing both overall survival (*Sirt2*^−/−^*; p53*^−/−^: HFD vs. ctrl *p* < 0.0001) and tumor incidence (*Sirt2*^−/−^*; p53*^−/−^: HFD vs. ctrl *p* < 0.0001) suggesting that SIRT2 exhibits tumor suppressive properties in this context as well (Figures 4A,B). However, a different response was observed in the *p53* heterozygous background (Figures 4C,D). Although there was a trend toward decreased survival in the *Sirt2*^−/−^*; p53*^+/−^ mice under HFD, they seemed to be partially protected as the decrease in tumor incidence did not reach statistical significance (*Sirt2*^−/−^*; p53*^+/−^: HFD vs. ctrl survival *p* = 0.01 / tumor incidence *p* = 0.1). The protective effect of *Sirt2* deletion in the *p53* heterozygous background was further supported by the observation that HFD S*irt2*^−/−^*; p53*^+/−^ mice, relative to *p53*^+/−^ mice, exhibited increased survival (HFD: *Sirt2*^−/−^*; p53*^+/−^ vs. *p53*^+/−^*p* = 0.03) and decreased tumor incidence (HFD: *Sirt2*^−/−^*; p53*^+/−^ vs. *p53*^+/−^ *p* = 0.01).

**Figure 5:**
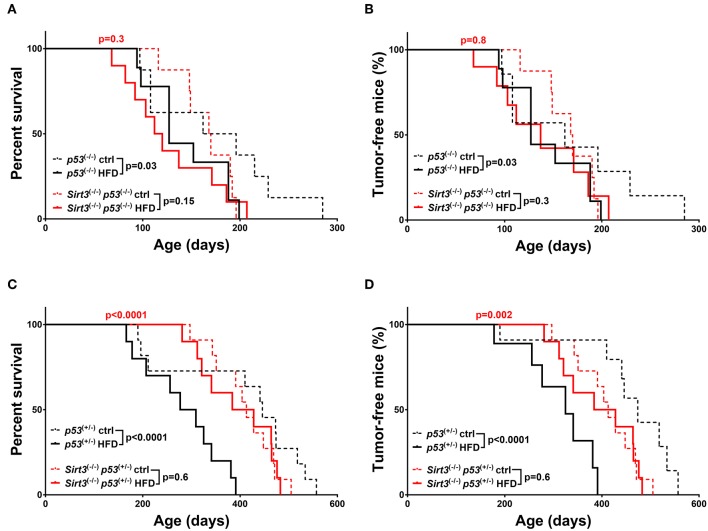
*Sirt3* deletion exhibited a protective effect against HFD-induced tumorigenesis. Kaplan-Meier curves show overall survival **(A,C)** and tumor free survival **(B,D)**. *Sirt3*^−/−^*; p53*^−/−^ (red lines), and *p53*^−/−^ (black lines) mice **(A,B)**, *Sirt3*^−/−^*; p53*^+/−^ (red lines), and *p53*^+/−^ (black lines) mice **(C,D)** were included in the analysis. Mice were either fed a high fat diet (HFD) or fed *ad libitum* control diet. Solid lines represent mice in the HFD groups, whereas control diet groups are represented in dotted lines. The red *p*-values on the top of each curve represent the statistical difference in survival and tumor incidence between HFD *Sirt3*^−/−^ mice, and HFD *Sirt3*^+/+^ mice with similar *p53 g*enotypes. All *p*-values are calculated using the log rank test.

With regards to the other sirtuin family member, *Sirt3* deletion resulted in decreased HFD-induced tumorigenesis, in both *p53* backgrounds, supporting a tumor promoting role for SIRT3 in this context. In a *p53* null background, *Sirt3*^−/−^ mice fed a HFD did not show statistically significant decrease in survival (*Sirt3*^−/−^*; p53*^−/−^: HFD vs. ctrl *p* = 0.15) and tumor incidence (*Sirt3*^−/−^*; p53*^−/−^: HFD vs. ctrl *p* = 0.3) as compared to *Sirt3*^+/+^ mice (Figures 5A,B). The protective effect of *Sirt3* loss was even more pronounced in the *p53* heterozygous mice (Figures 5C,D). In contrast to the *p53*^+/−^ mice, HFD did not decrease survival of *Sirt3*^−/−^*p53*^+/−^ mice (*p53*^+/−^: HFD vs. ctrl *p* < 0.0001 / *Sirt3*^−/−^*; p53*^+/−^: HFD vs. ctrl *p* = 0.6). Consistently, HFD did not affect tumor incidence in mice with *Sirt3* deletion (*p53*^+/−^: HFD vs. ctrl *p* < 0.0001/*Sirt3*^−/−^*; p53*^+/−^: HFD vs. ctrl *p* = 0.6). As a result, *Sirt3*^−/−^*; p53*^+/−^ mice exhibited significant protection against the tumor promoting effects of HFD compared to *p53*^+/−^ mice. This is supported by the increased overall (HFD: *p53*^+/−^ vs. *Sirt3*^−/−^*; p53*^+/−^*p* < 0.0001) and median (HFD: 293 days *p53*^+/−^ vs. 406 days *Sirt3*^−/−^*; p53*^+/−^) survival, as well as decreased tumor incidence (HFD: *p53*^+/−^ vs. *Sirt3*^−/−^*; p53*^+/−^*p* = 0.002) (Figures 5C,D and Supplementary Table 1). Taken together, these results highlight the different roles played by the two sirtuin family members in HFD-induced tumorigenesis. While SIRT2 maintains its tumor suppressive properties (at least in a *p53* null background), SIRT3 switches to an oncogenic role in mice fed a HFD suggesting that SIRT3 inhibition renders mice resistant to HFD-induced tumorigenesis.

### Tumor Spectrum Analysis in Sirt2 and Sirt3 Null Mice Upon CR and HFD

**Figure 6:**
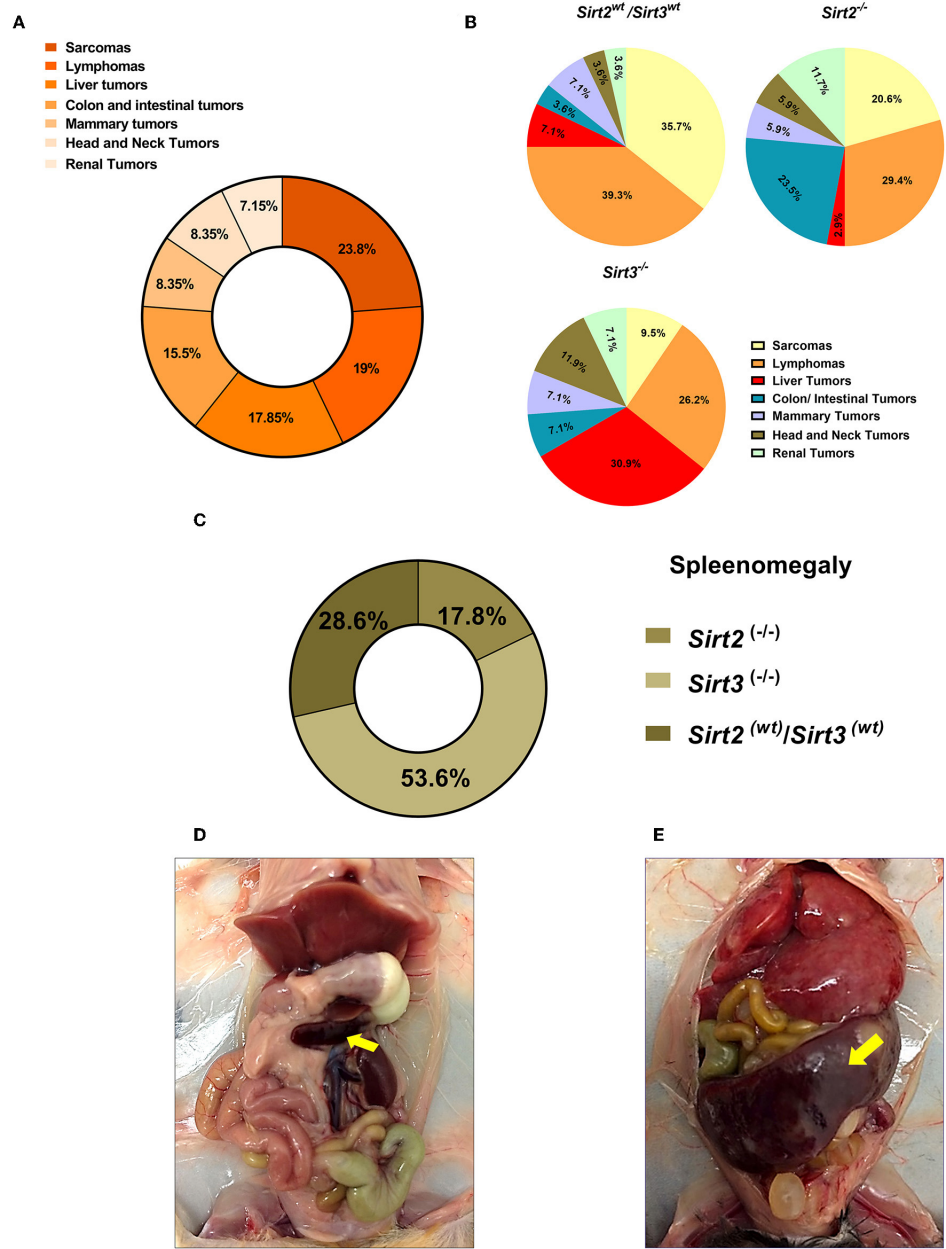
Spectrum of tumors developed in the *Sirt2* and *Sirt3*-deficient mice. **(A)** Percentages of different tumor types refer to proportion of all tumors reported, with sarcoma and lymphoma being the most prominent tumors reported. **(B)** Relative frequencies of the different types of tumors detected in *Sirt2*^*wt*^*/Sirt3*^*wt*^, *Sirt2*^−/−^, and *Sirt3*^−/−^ mice, regardless of the dietary group, are displayed by pie charts. **(C)** 26 mice with splenomegaly were found in this study. Majority of cases (~54%) were reported in *Sirt3* knockout mice. **(D,E)** Representative pictures of a mouse with normal spleen size **(D)** and a mouse with splenomegaly **(E)** are shown.

We noticed that *p53*-related tumors were the major tumors developed in mice of our study, with sarcomas and lymphomas representing more than 50% of all tumors (Figure 6A). Interestingly, we observed strong association between the loss of *Sirt2*/*Sirt3* and certain tumors (Figure 6B and Supplementary Table 2). Under different dietary regimens, *Sirt3* deficient mice experienced the highest incidence rate of liver tumors and the lowest incidence of sarcomas, either in *p53*^+/−^ or *p53*^−/−^ background, compared to the other groups. On the other hand, colon/intestinal and renal tumors were increased in *Sirt2* knockout mice as compared to other genotypes. Moreover, we observed an increased incidence of splenomegaly in mice with *Sirt3* deletion, when compared to the other genotypes, regardless of *p53* status (Figures 6C–E).

## Discussion

SIRT2 is the least comprehensively studied member of mammalian sirtuins, especially for its role in the context of calorie restriction. The results from this study provide evidence, for the first time, to support a role for SIRT2 in mediating the protective effects of CR against tumorigenesis. Although controversial roles for SIRT2 in cancer have been described (32), our data show that SIRT2 exhibits tumor suppressive properties upon calorie restriction. Interestingly, this happened despite the decreased overall weight of calorie restricted *Sirt2*^−/−^ mice as compared to *Sirt2* wild-type mice suggesting that SIRT2-directed downstream signaling is impaired. The observed effect is consistent with previous studies showing that SIRT2 functions as a tumor suppressor (28, 33–35). *Sirt2* loss negates the protective effect of CR on tumor development in a *p53* mouse model and, therefore, unraveling downstream pathways regulated by SIRT2 in this context is warranted. It is worth mentioning that despite that several SRT2 deacetylation targets have been reported, a comprehensive analysis of SIRT2 targets using unbiased high throughput approaches is currently missing. We are in the process of completing such an analysis (data not show), which will enable to associate SIRT2 targets with molecular pathways related to CR and provide mechanistic insights. Very recently, Diaz-Ruiz et al. showed that upregulation of NQO1, an essential NADH-dehydrogenase that mediates redox control of metabolic homeostasis, mimicks aspects of CR including protection against carcinogenesis in mice (36). Of note, our latest study showed that NQO1 interacts with and activates SIRT2 in an NAD-dependent manner (37). Therefore, it is intriguing to further investigate whether SIRT2 activation mediates the CR-mimetic effects of NQO1 overexpression or whether SIRT2 overexpression alone can mimic the beneficial effects exerted by CR on cancer prevention, further emphasizing the potential for exploiting SIRT2 as a target for cancer prevention.

SIRT3 has been previously described to mediate CR-related health benefits (25, 30, 38). However, the involvment of SIRT3 in regulating diet-dependent tumor development is obscure. By using a *p53* mouse model, here we show that SIRT3 plays a prominent role in HFD-induced tumorigenesis. Notably, *Sirt3* deficient mice were resistant to the tumor promoting effect of HFD. Although it has been previously shown that *Sirt3* loss in mice on a high-fat diet accelerates obesity, insulin resistance, hyperlipidemia, and steatohepatitis (39), and SIRT3 preserves heart function and capillary density (40), our results show that *Sirt3* loss increases survival and decreases tumor incidence upon HFD. Therefore, it can be proposed that SIRT3 exhibits oncogenic properties in the context of HFD-induced tumorigenesis and SIRT3 inhibition might be suggested as a strategy to mitigate the tumor promoting effects of HFD. The protective effect of *Sirt3* loss, especially in the *p53*^+/−^ background, was observed while mice gained weight as a response to the HFD. Therefore, it is reasonable to suggest that SIRT3 functions previously shown to support its role as an oncogene might be involved. In this regard, regulation of ROS production and mitochondrial metabolism (41–43) might provide the mechanistic link to explain the protective effect of SIRT3 inhibition in HFD-induced tumorigenesis. Of note, our unbiased proteomics-based analysis to detect proteins with increased acetylated levels in *Sirt3*^−/−^ liver tissue as compared to *Sirt3*^+/+^ tissue revealed that fatty acid metabolic process was the most significantly enriched pathway using (Supplementary Figure 1). This is consistent with previous studies showing that SIRT3 regulates fatty acid oxidation (25, 44), while Perturbations in fatty acid metabolism have been associated with cancer (45). More importantly, regulation of fatty acid metabolism is critical especially in mice fed a HFD diet. Therefore, it is possible that dysregulation of this pathway in *Sirt3*^−/−^ mice might provide some mechanistic insights to explain the observed resistance to HFD-induced tumorigenesis.

Both *p53*^−/−^ and *p53*^+/−^ mice were included in our analysis to address whether p53-dependent functions are involved. Upon CR, *Sirt2* deletion abolished the protective effect in both genotypes, supporting a critical role for SIRT2 upon CR. In the HFD-fed mice, *Sirt3* deletion rendered mice more resistant to HFD-induced tumorigenesis in both *p53* genotypes, suggesting a role for SIRT3 in this context. p53 has been previously shown to regulate metabolism and mitochondrial function (46–48). The observed phenotypes in mice lacking *p53* favor a role for these sirtuin family members involving p53-independent mechanisms. Although some responses seem to be stronger in the *p53* heterozygous as compared to the *p53* homozygous knockout mice, we believe that this might due to the different total time that the mice have been exposed to the two diets. In this regard, *p53*^+/−^ mice develop tumors later in life compared to the *p53*^−/−^ mice which allowed 2 cycles of feeding with the different diets as opposed to one cycle in the *p53*^−/−^ mice.

**Figure 7:**
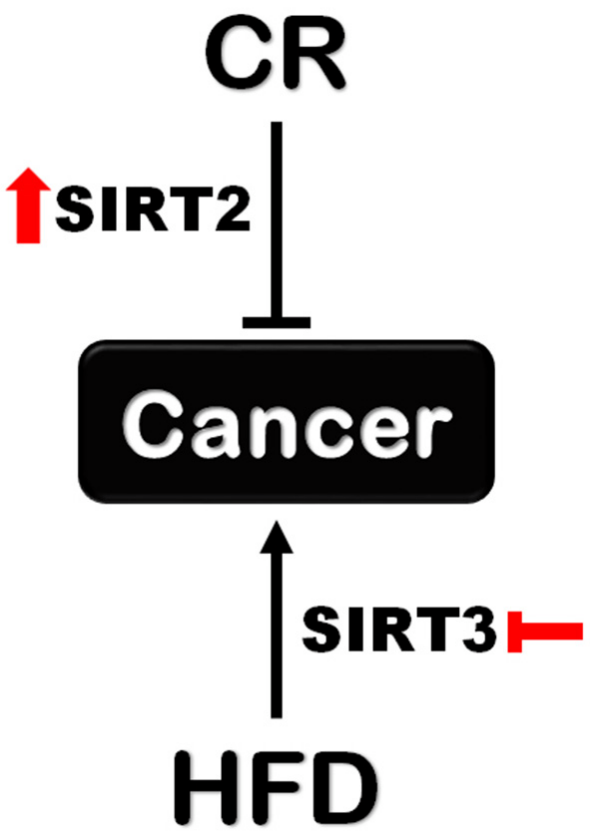
Different roles for SIRT2 and SIRT3 in tumorigenesis upon CR and HFD. *Sirt2* is indispensable for the protective effect of CR against tumorigenesis supporting a cancer-preventive role for SIRT2 activation. On the other hand, *Sirt3* loss renders mice resistant to HFD-induced tumorigenesis which supports the beneficial effect of SIRT3 inhibition in this context.

Collectively, our data support new context dependent roles for SIRT2 and SIRT3 in regulating the effects of CR and HFD on tumorigenesis. Given the lack of experimental data to address the contribution of situin family members other than SIRT1, our results reignite interest regarding the contribution of sirtuins under these specific metabolic conditions. Therefore, SIRT2 activation upon CR and SIRT3 inhibition upon HFD could be explored as new strategies to enhance CR-induced cancer prevention and decrease HFD-induced tumor development, respectively (Figure 7). Future studies focusing on unraveling downstream pathways and mechanisms hold the promise for identifying novel interventions to either mimic the cancer-preventive effect of CR or protect against HFD-induced tumorigenesis.

## Data Availability Statement

All datasets generated for this study are included in the article/Supplementary Material.

## Ethics Statement

The animal study was reviewed and approved by Institutional Animal Care and Use Committee (IACUC) Northwestern University.

## Author Contributions

MA and CO'C performed experiments and collected data. HJ maintained the mice colonies. EC helped with tissue collection and tissue sample analysis. MA performed statistical analyses. CO'C and AV designed the study. MA and AV prepared the manuscript.

### Conflict of Interest

The authors declare that the research was conducted in the absence of any commercial or financial relationships that could be construed as a potential conflict of interest.
