# Supplementary material for: Context-Dependent Roles for SIRT2 and SIRT3 in Tumor Development Upon Calorie Restriction or High Fat Diet
Source: Front Oncol. 2020 Jan 8;9:1462. doi: 10.3389/fonc.2019.01462 (PMC6960403; doi:10.3389/fonc.2019.01462)
Supplement: Supplementary file 1 [file Data_Sheet_1.PDF]

Context-dependent roles for SIRT2 and SIRT3 in tumor development upon calorie restriction or high fat diet

Mohamed A. Ahmed[1,3], Carol O'Callaghan[1], Elliot D. Chang[1], Haiyan Jiang[1], and Athanassios Vassilopoulos[1, 2]

[1] Department of Radiation Oncology, Feinberg School of Medicine, Northwestern University, Chicago, IL, 60611, USA.
[2] Robert H. Lurie Comprehensive Cancer Center, Feinberg School of Medicine, Northwestern University, Chicago, IL, 60611, USA.
[3] Radiation Biology Department, National Center for Radiation Research and Technology, Cairo, Egypt.

Supplementary Tables and Figures:

Diet Group		Ctrl			CR			HFD		
Genotype		***Sirt2***$^{+/+}$ ***Sirt3***$^{+/+}$	***Sirt2***$^{-/-}$ ***Sirt3***$^{+/+}$	***Sirt2***$^{+/+}$ ***Sirt3***$^{-/-}$	***Sirt2***$^{+/+}$ ***Sirt3***$^{+/+}$	***Sirt2***$^{-/-}$ ***Sirt3***$^{+/+}$	***Sirt2***$^{+/+}$ ***Sirt3***$^{-/-}$	***Sirt2***$^{+/+}$ ***Sirt3***$^{+/+}$	***Sirt2***$^{-/-}$ ***Sirt3***$^{+/+}$	***Sirt2***$^{+/+}$ ***Sirt3***$^{-/-}$
p53 (+/-)	**Sex (M:F)**	6:5	6:5	5:6	5:6	6:5	5:6	5:6	5:5	5:7
	**Median age (days)**	446	471	413	550	309	415	293	353	406
	**Hazard Ratio (HR)**	-	-	-	0.46	1.67	0.65	4.01	1.58	1.13
p53 (-/-)	**Sex (M:F)**	6:6	6:5	5:4	5:7	5:6	5:5	6:5	5:6	7:4
	**Median age (days)**	179	181.5	169	189	148	158	139.5	111	112
	**Hazard Ratio (HR)**	-	-	-	0.55	1.16	0.45	1.58	3.55	1.24

Supplementary Table (1): Diet-induced changes in median survival and hazard ratios of tumor incidence. The hazard ratios, indicating changes in tumor incidence rate compared to control diet group, were calculated from the slopes of tumor-free survival curves (Fig. 2-5) and estimated based on log-rank test.

Diet	Genotype		Gender	Age (days)	Tumors observed							
	p53	*Sirt2/3*			Sarcoma	Lymphomas	Liver	Colon/Intestinal	Mammary	Head and Neck	Renal	Spleenomegaly
CTRL	*p53 (-/-)*	wt	Male	229		x						
CTRL	*p53 (-/-)*	wt	Male	162*				***found dead / macroscopic analysis for tumors was not possible***				
CTRL	*p53 (-/-)*	wt	Male	215								
CTRL	*p53 (-/-)*	wt	Male	196	x	x						
CTRL	*p53 (-/-)*	wt	Male	108		x						
CTRL	*p53 (-/-)*	wt	Female	285	x							
CTRL	*p53 (-/-)*	wt	Female	248		x						
CTRL	*p53 (-/-)*	wt	Female	49								
CTRL	*p53 (-/-)*	wt	Female	97								
CTRL	*p53 (-/-)*	wt	Female	222	x							
CTRL	*p53 (-/-)*	*Sirt2 (-/-)*	Male	181								
CTRL	*p53 (-/-)*	*Sirt2 (-/-)*	Male	198				x				
CTRL	*p53 (-/-)*	*Sirt2 (-/-)*	Male	107								
CTRL	*p53 (-/-)*	*Sirt2 (-/-)*	Male	220								
CTRL	*p53 (-/-)*	*Sirt2 (-/-)*	Male	102								
CTRL	*p53 (-/-)*	*Sirt2 (-/-)*	Male	182		x						
CTRL	*p53 (-/-)*	*Sirt2 (-/-)*	Female	141				x				
CTRL	*p53 (-/-)*	*Sirt2 (-/-)*	Female	186								
CTRL	*p53 (-/-)*	*Sirt2 (-/-)*	Female	172	x							
CTRL	*p53 (-/-)*	*Sirt2 (-/-)*	Female	244				x		x		
CTRL	*p53 (-/-)*	*Sirt2 (-/-)*	Female	221								
CTRL	*p53 (-/-)*	*Sirt3 (-/-)*	Male	116								
CTRL	*p53 (-/-)*	*Sirt3 (-/-)*	Male	187		x	x					
CTRL	*p53 (-/-)*	*Sirt3 (-/-)*	Male	168	x							
CTRL	*p53 (-/-)*	*Sirt3 (-/-)*	Male	190								
CTRL	*p53 (-/-)*	*Sirt3 (-/-)*	Male	148	x							
CTRL	*p53 (-/-)*	*Sirt3 (-/-)*	Female	170								
CTRL	*p53 (-/-)*	*Sirt3 (-/-)*	Female	192								
CTRL	*p53 (-/-)*	*Sirt3 (-/-)*	Female	196*				***found dead / macroscopic analysis for tumors was not possible***				
CTRL	*p53 (-/-)*	*Sirt3 (-/-)*	Female	149						x		
CTRL	*p53 (+/-)*	wt	Male	211								
CTRL	*p53 (+/-)*	wt	Male	474								x
CTRL	*p53 (+/-)*	wt	Male	190								
CTRL	*p53 (+/-)*	wt	Male	518								x
CTRL	*p53 (+/-)*	wt	Male	410*				***found dead / macroscopic analysis for tumors was not possible***				
CTRL	*p53 (+/-)*	wt	Female	196								
CTRL	*p53 (+/-)*	wt	Female	534								
CTRL	*p53 (+/-)*	wt	Female	441*				***found dead / macroscopic analysis for tumors was not possible***				
CTRL	*p53 (+/-)*	wt	Female	446								
CTRL	*p53 (+/-)*	wt	Female	473								
CTRL	*p53 (+/-)*	*Sirt2 (-/-)*	Male	186								
CTRL	*p53 (+/-)*	*Sirt2 (-/-)*	Male	265								
CTRL	*p53 (+/-)*	*Sirt2 (-/-)*	Male	570	x			x				
CTRL	*p53 (+/-)*	*Sirt2 (-/-)*	Male	521		x						
CTRL	*p53 (+/-)*	*Sirt2 (-/-)*	Male	563						x		
CTRL	*p53 (+/-)*	*Sirt2 (-/-)*	Male	529								
CTRL	*p53 (+/-)*	*Sirt2 (-/-)*	Female	505				x				x
CTRL	*p53 (+/-)*	*Sirt2 (-/-)*	Female	557*				***found dead / macroscopic analysis for tumors was not possible***				
CTRL	*p53 (+/-)*	*Sirt2 (-/-)*	Female	421								
CTRL	*p53 (+/-)*	*Sirt2 (-/-)*	Female	301					x			
CTRL	*p53 (+/-)*	*Sirt2 (-/-)*	Female	315								x
CTRL	*p53 (+/-)*	*Sirt3 (-/-)*	Male	343*				***found dead / macroscopic analysis for tumors was not possible***				
CTRL	*p53 (+/-)*	*Sirt3 (-/-)*	Male	471			x					
CTRL	*p53 (+/-)*	*Sirt3 (-/-)*	Male	391	x							
CTRL	*p53 (+/-)*	*Sirt3 (-/-)*	Male	428*				***found dead / macroscopic analysis for tumors was not possible***				
CTRL	*p53 (+/-)*	*Sirt3 (-/-)*	Male	448			x					x
CTRL	*p53 (+/-)*	*Sirt3 (-/-)*	Female	404*				***found dead / macroscopic analysis for tumors was not possible***				
CTRL	*p53 (+/-)*	*Sirt3 (-/-)*	Female	413*				***found dead / macroscopic analysis for tumors was not possible***				
CTRL	*p53 (+/-)*	*Sirt3 (-/-)*	Female	405	x		x					
CTRL	*p53 (+/-)*	*Sirt3 (-/-)*	Female	297								x
CTRL	*p53 (+/-)*	*Sirt3 (-/-)*	Female	469					x			x
CTRL	*p53 (+/-)*	*Sirt3 (-/-)*	Female	351							x	

CR	*p53 (-/-)*	wt	Male	266								**x**
CR	*p53 (-/-)*	wt	Male	129								
CR	*p53 (-/-)*	wt	Male	189								
CR	*p53 (-/-)*	wt	Male	120		**x**						
CR	*p53 (-/-)*	wt	Male	225	**x**							
CR	*p53 (-/-)*	wt	Female	189							**x**	**x**
CR	*p53 (-/-)*	wt	Female	104						**x**		
CR	*p53 (-/-)*	wt	Female	369								
CR	*p53 (-/-)*	wt	Female	166	**x**							
CR	*p53 (-/-)*	wt	Female	294					**x**			
CR	*p53 (-/-)*	wt	Female	262								
CR	*p53 (-/-)*	wt	Female	283								
CR	*p53 (-/-)*	*Sirt2 (-/-)*	Male	197								
CR	*p53 (-/-)*	*Sirt2 (-/-)*	Male	131	**x**							
CR	*p53 (-/-)*	*Sirt2 (-/-)*	Male	148		**x**						
CR	*p53 (-/-)*	*Sirt2 (-/-)*	Male	237								
CR	*p53 (-/-)*	*Sirt2 (-/-)*	Male	85		**x**						
CR	*p53 (-/-)*	*Sirt2 (-/-)*	Female	50*	***found dead / macroscopic analysis for tumors was not possible***							
CR	*p53 (-/-)*	*Sirt2 (-/-)*	Female	227							**x**	
CR	*p53 (-/-)*	*Sirt2 (-/-)*	Female	218		**x**						**x**
CR	*p53 (-/-)*	*Sirt2 (-/-)*	Female	49								
CR	*p53 (-/-)*	*Sirt2 (-/-)*	Female	207								
CR	*p53 (-/-)*	*Sirt3 (-/-)*	Male	124		**x**						
CR	*p53 (-/-)*	*Sirt3 (-/-)*	Male	139								
CR	*p53 (-/-)*	*Sirt3 (-/-)*	Male	133*	***found dead / macroscopic analysis for tumors was not possible***							
CR	*p53 (-/-)*	*Sirt3 (-/-)*	Male	113								**x**
CR	*p53 (-/-)*	*Sirt3 (-/-)*	Female	90								
CR	*p53 (-/-)*	*Sirt3 (-/-)*	Female	257					**x**			
CR	*p53 (-/-)*	*Sirt3 (-/-)*	Female	285*	***found dead / macroscopic analysis for tumors was not possible***							
CR	*p53 (-/-)*	*Sirt3 (-/-)*	Female	158					**x**			
CR	*p53 (-/-)*	*Sirt3 (-/-)*	Female	224						**x**		
CR	*p53 (-/-)*	*Sirt3 (-/-)*	Female	243								
CR	*p53 (+/-)*	wt	Male	564*	***found dead / macroscopic analysis for tumors was not possible***							
CR	*p53 (+/-)*	wt	Male	555				**x**				
CR	*p53 (+/-)*	wt	Male	409								
CR	*p53 (+/-)*	wt	Male	603								
CR	*p53 (+/-)*	wt	Male	550	**x**							
CR	*p53 (+/-)*	wt	Female	62								
CR	*p53 (+/-)*	wt	Female	507								
CR	*p53 (+/-)*	wt	Female	177								
CR	*p53 (+/-)*	wt	Female	437	**x**							
CR	*p53 (+/-)*	wt	Female	657								
CR	*p53 (+/-)*	*Sirt2 (-/-)*	Male	398							**x**	
CR	*p53 (+/-)*	*Sirt2 (-/-)*	Male	64								
CR	*p53 (+/-)*	*Sirt2 (-/-)*	Male	436*	***found dead / macroscopic analysis for tumors was not possible***							
CR	*p53 (+/-)*	*Sirt2 (-/-)*	Male	474								
CR	*p53 (+/-)*	*Sirt2 (-/-)*	Female	544		**x**						**x**
CR	*p53 (+/-)*	*Sirt2 (-/-)*	Female	143								
CR	*p53 (+/-)*	*Sirt2 (-/-)*	Female	309								
CR	*p53 (+/-)*	*Sirt2 (-/-)*	Female	304*	***found dead / macroscopic analysis for tumors was not possible***							
CR	*p53 (+/-)*	*Sirt2 (-/-)*	Female	553	**x**			**x**				
CR	*p53 (+/-)*	*Sirt3 (-/-)*	Male	388			**x**					**x**
CR	*p53 (+/-)*	*Sirt3 (-/-)*	Male	361								
CR	*p53 (+/-)*	*Sirt3 (-/-)*	Male	409								**x**
CR	*p53 (+/-)*	*Sirt3 (-/-)*	Male	415			**x**					**x**
CR	*p53 (+/-)*	*Sirt3 (-/-)*	Female	81								
CR	*p53 (+/-)*	*Sirt3 (-/-)*	Female	466		**x**						
CR	*p53 (+/-)*	*Sirt3 (-/-)*	Female	478								
CR	*p53 (+/-)*	*Sirt3 (-/-)*	Female	373			**x**					**x**
CR	*p53 (+/-)*	*Sirt3 (-/-)*	Female	370		**x**	**x**					
CR	*p53 (+/-)*	*Sirt3 (-/-)*	Female	272		**x**						

HFD	*p53 (-/-)*	wt	Male	188		x						
HFD	*p53 (-/-)*	wt	Male	94								
HFD	*p53 (-/-)*	wt	Male	98								
HFD	*p53 (-/-)*	wt	Male	152*	***found dead / macroscopic analysis for tumors was not possible***							
HFD	*p53 (-/-)*	wt	Female	123	x							
HFD	*p53 (-/-)*	wt	Female	199					x			
HFD	*p53 (-/-)*	wt	Female	127		x						
HFD	*p53 (-/-)*	wt	Female	217		x	x					x
HFD	*p53 (-/-)*	wt	Female	127		x						
HFD	*p53 (-/-)*	*Sirt2 (-/-)*	Male	61	x	x						x
HFD	*p53 (-/-)*	*Sirt2 (-/-)*	Male	53								
HFD	*p53 (-/-)*	*Sirt2 (-/-)*	Male	111								
HFD	*p53 (-/-)*	*Sirt2 (-/-)*	Male	132								
HFD	*p53 (-/-)*	*Sirt2 (-/-)*	Male	139								
HFD	*p53 (-/-)*	*Sirt2 (-/-)*	Female	141		x						
HFD	*p53 (-/-)*	*Sirt2 (-/-)*	Female	105	x				x			
HFD	*p53 (-/-)*	*Sirt2 (-/-)*	Female	92								
HFD	*p53 (-/-)*	*Sirt2 (-/-)*	Female	155*	***found dead / macroscopic analysis for tumors was not possible***							
HFD	*p53 (-/-)*	*Sirt2 (-/-)*	Female	179								
HFD	*p53 (-/-)*	*Sirt2 (-/-)*	Female	110								
HFD	*p53 (-/-)*	*Sirt3 (-/-)*	Male	82								
HFD	*p53 (-/-)*	*Sirt3 (-/-)*	Male	120								
HFD	*p53 (-/-)*	*Sirt3 (-/-)*	Male	103							x	
HFD	*p53 (-/-)*	*Sirt3 (-/-)*	Male	68								
HFD	*p53 (-/-)*	*Sirt3 (-/-)*	Male	171						x		
HFD	*p53 (-/-)*	*Sirt3 (-/-)*	Male	207								
HFD	*p53 (-/-)*	*Sirt3 (-/-)*	Male	112								
HFD	*p53 (-/-)*	*Sirt3 (-/-)*	Female	92*	***found dead / macroscopic analysis for tumors was not possible***							
HFD	*p53 (-/-)*	*Sirt3 (-/-)*	Female	137*	***found dead / macroscopic analysis for tumors was not possible***							
HFD	*p53 (-/-)*	*Sirt3 (-/-)*	Female	179						x		
HFD	*p53 (-/-)*	*Sirt3 (-/-)*	Female	186								
HFD	*p53 (+/-)*	wt	Male	309								
HFD	*p53 (+/-)*	wt	Male	228	x	x						
HFD	*p53 (+/-)*	wt	Male	207*	***euthanized by vet due to fight wounds***							
HFD	*p53 (+/-)*	wt	Male	341*	***found dead / macroscopic analysis for tumors was not possible***							
HFD	*p53 (+/-)*	wt	Male	277				x				
HFD	*p53 (+/-)*	wt	Female	381*	***found dead / macroscopic analysis for tumors was not possible***							
HFD	*p53 (+/-)*	wt	Female	178*	***found dead / macroscopic analysis for tumors was not possible***							
HFD	*p53 (+/-)*	wt	Female	391	x							
HFD	*p53 (+/-)*	wt	Female	166								
HFD	*p53 (+/-)*	wt	Female	325		x						x
HFD	*p53 (+/-)*	wt	Female	256			x					
HFD	*p53 (+/-)*	*Sirt2 (-/-)*	Male	334				x				
HFD	*p53 (+/-)*	*Sirt2 (-/-)*	Male	157								
HFD	*p53 (+/-)*	*Sirt2 (-/-)*	Male	254								
HFD	*p53 (+/-)*	*Sirt2 (-/-)*	Male	468		x	x					x
HFD	*p53 (+/-)*	*Sirt2 (-/-)*	Male	450								
HFD	*p53 (+/-)*	*Sirt2 (-/-)*	Female	340								
HFD	*p53 (+/-)*	*Sirt2 (-/-)*	Female	493								
HFD	*p53 (+/-)*	*Sirt2 (-/-)*	Female	353*	***found dead / macroscopic analysis for tumors was not possible***							
HFD	*p53 (+/-)*	*Sirt2 (-/-)*	Female	426		x		x				
HFD	*p53 (+/-)*	*Sirt2 (-/-)*	Female	409	x						x	
HFD	*p53 (+/-)*	*Sirt3 (-/-)*	Male	460*	***found dead / macroscopic analysis for tumors was not possible***							
HFD	*p53 (+/-)*	*Sirt3 (-/-)*	Male	312*	***found dead / macroscopic analysis for tumors was not possible***							
HFD	*p53 (+/-)*	*Sirt3 (-/-)*	Male	465		x		x				
HFD	*p53 (+/-)*	*Sirt3 (-/-)*	Male	321				x				
HFD	*p53 (+/-)*	*Sirt3 (-/-)*	Male	428		x	x					x
HFD	*p53 (+/-)*	*Sirt3 (-/-)*	Female	513		x	x				x	x
HFD	*p53 (+/-)*	*Sirt3 (-/-)*	Female	464		x	x					x
HFD	*p53 (+/-)*	*Sirt3 (-/-)*	Female	483		x						x
HFD	*p53 (+/-)*	*Sirt3 (-/-)*	Female	384			x	x				
HFD	*p53 (+/-)*	*Sirt3 (-/-)*	Female	476		x	x					x

<u>Supplementary Table (2):</u> Tumors developed in the *Sirt2^wt^/Sirt3^wt^*, *Sirt2^-/-^* and *Sirt3^-/-^* mice. Table shows diet, genotype, gender, age and types of tumors for all mice included in the study.

A

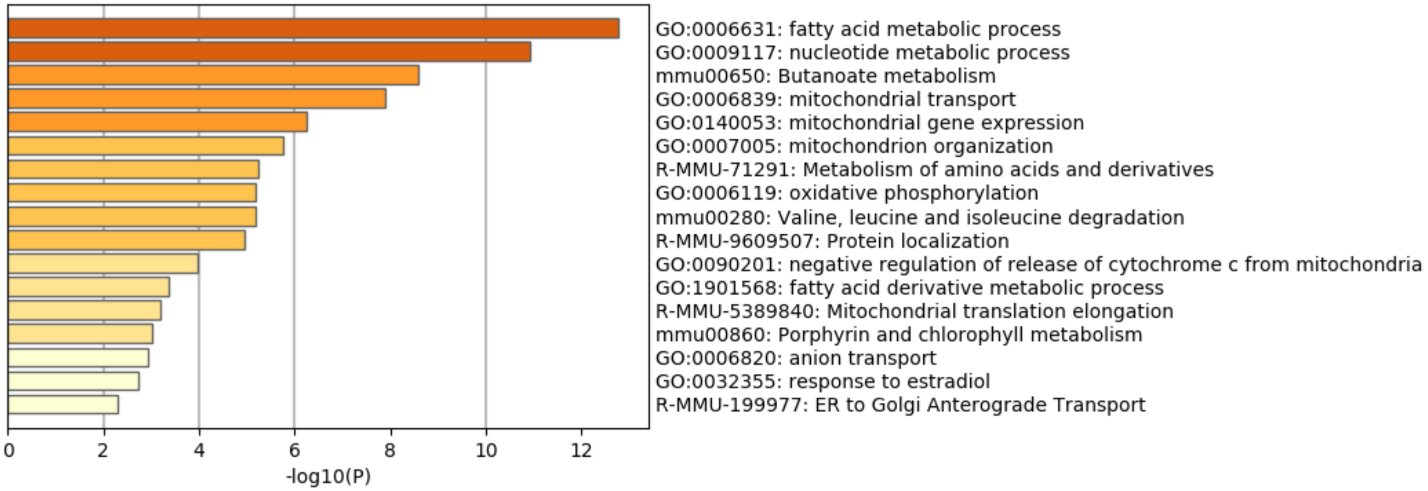

B

Acadl|Aldh3a2|Cpt2|Ggt1|Anxa1|Echdc2|Mlycd|Them4|Acox3|Hadha|Cyp2c50|Acsm1|Acad9|Hadhb|Acsm2|Acsf3|Acsm5|Cyp2c68

Supplementary Figure 1. Proteomics based approach to analyze the acetylome in *Sirt3*$^{+/+}$ and *Sirt3*$^{-/-}$ mice. Proteins with increased acetylation in *Sirt3*$^{-/-}$ liver tissue as compared to *Sirt3*$^{+/+}$ tissue were detected by mass spectrometry following immunoprecipitation with an anti-acetyllysine antibody (ICP0388; ImmunoChem Pharmaceuticals, Inc.). (A) Pathway analysis was performed using Metascape. Bar graph of enriched terms across input protein list, colored by p-values is shown. (B) List of proteins with increased acetylation in *Sirt3*$^{-/-}$ liver tissue as compared to *Sirt3*$^{+/+}$ tissue included in the Fatty acid metabolic process cluster is shown.
